# Osteoarthritis year in review: genetics, genomics, epigenetics

**DOI:** 10.1016/j.joca.2021.11.004

**Published:** 2022-02

**Authors:** D.A. Young, M.J. Barter, J. Soul

**Affiliations:** Skeletal Research Group, Biosciences Institute, Newcastle University, Central Parkway, Newcastle upon Tyne, NE1 3BZ, UK

**Keywords:** Osteoarthritis, Genetics, Genomics, Epigenetics, microRNA, circRNA, lncRNA

## Abstract

**Objective:**

In this review, we have highlighted the advances over the past year in genetics, genomics and epigenetics in the ﬁeld of osteoarthritis (OA).

**Methods:**

A literature search of PubMed was performed using the criteria: “osteoarthritis” and one of the following terms “genetic(s), genomic(s), epigenetic(s), polymorphism, noncoding ribonucleic acid (RNA), microRNA, long noncoding RNA, lncRNA, circular RNA, RNA sequencing (RNA-seq), single cell sequencing, transcriptomics, or deoxyribonucleic acid (DNA) methylation between April 01, 2020 and April 30, 2021.

**Results:**

In total we identiﬁed 765 unique publications, which eventually reduced to 380 of relevance to the field as judged by two assessors. Many of these studies included multiple search terms. We summarised advances relating to genetics, functional genetics, genomics and epigenetics, focusing on our personal key papers during the year.

**Conclusions:**

This year few studies have identiﬁed new genetic variants contributing to OA susceptibility, but a focus has been on refining risk loci or their functional validation. The use of new technologies together with investigating the cross-talk between multiple tissue types, greater sample sizes and/or better patient classification (OA subtypes) will continue to increase our knowledge of disease mechanisms and progress towards understanding and treating OA.

## Introduction

**Fig. 1:**
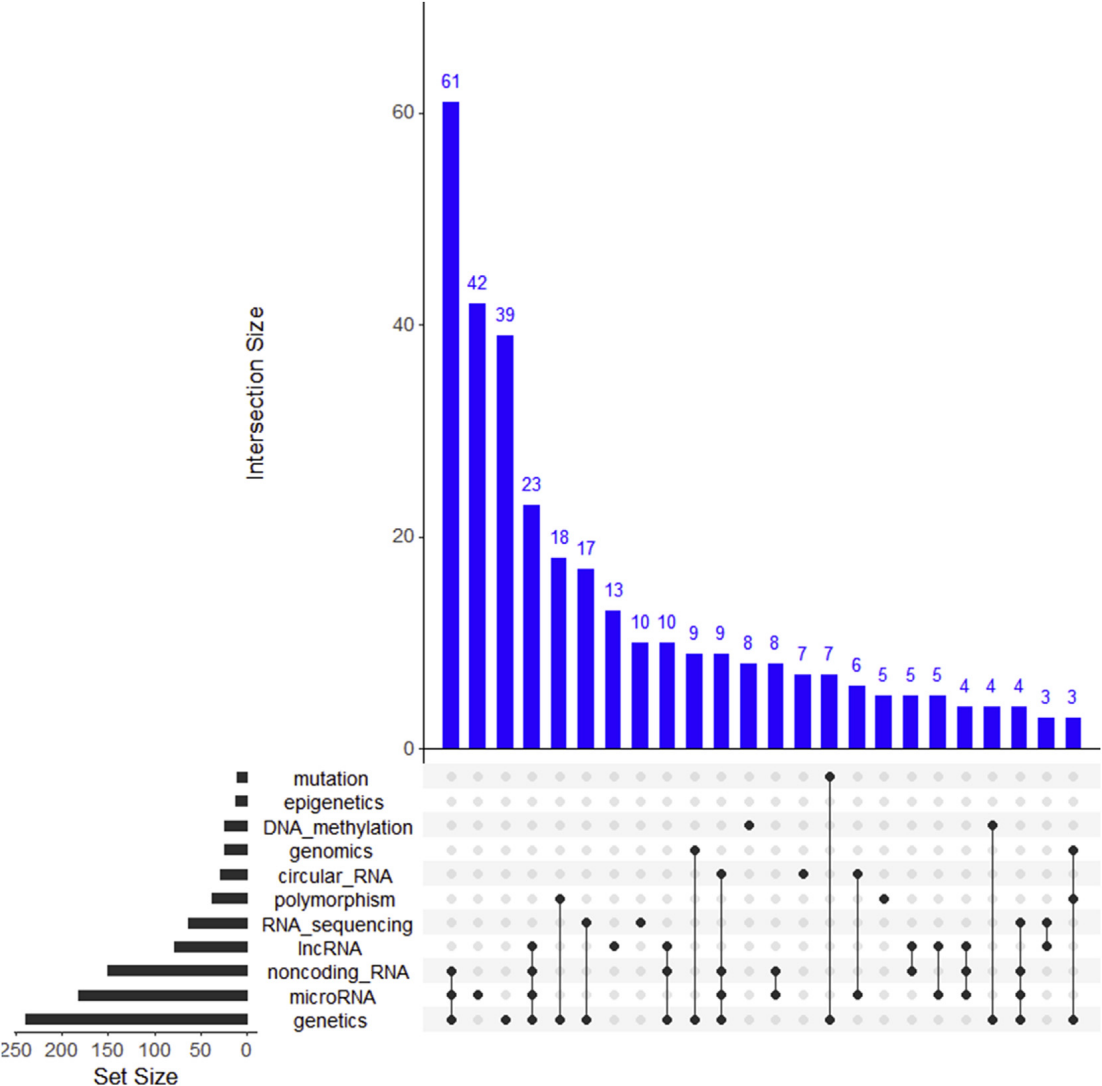
**Number of new publications indexed in PubMed with the defined search criteria after curation.** Search was undertaken between April 1st 2020 and April 30th 2021. Set Size defines the total number of publications with the matching search term + ‘osteoarthritis’ after curation. Main Upset plot shows number of publications (≥3) with overlapping search criteria. Plotted using UpSetR version 1.4.0^73^.

Osteoarthritis (OA) is the most prevalent musculoskeletal disease. The goal of this narrative review is to highlight key research studies published in OA genetics, genomics and epigenetics between April 1st 2020 and April 30th 2021. Using the defined search criteria we identified 765 articles, which when manually curated decreased to 380 deemed of relevance to the OA field (Supplementary File 1), many of which were identified by multiple search terms (Fig. 1). We also removed articles which contained potential irregularities identified in PubPeer (https://pubpeer.com/). Articles were chosen for discussion based on those we deemed to be novel, impactful to the field and fitted within the themes mentioned in this review.

## Genetics

This year saw a dearth of large genome-wide association studies (GWAS) for the major joints affected by OA, instead the focus was generally upon assessing previously identified genetic associations in differing ethnic populations (Table I). The growth differentiation factor 5 (*GDF5*) polymorphism rs143383 is reproducibly associated with OA, and this year was associated with OA-risk in the Kurdish population^1^. Oddly, in two meta-analyses examining the association of rs143383 with knee OA (KOA) in Asian individuals contradictory findings were observed^2^^,^^3^, while a new study in a Chinese Han population did find a link with KOA^4^. rs143383 is located in the 5′UTR of *GDF5* and is associated with differential allelic expression of the gene, via altered DNA-binding of several transcriptional repressors^5^. However, recent compelling data, including a study discussed in last year's review^6^, suggests that the OA-risk mediated by the *GDF* locus could be via the control of the growth factor expression by highly conserved distal regulatory elements, with comprehensive work characterizing the effects of the rs6060369 polymorphism in mouse knee cartilage and OA-development^7^.

**Table I:** Studies from April 12,020 to April 30 2021 examining genetic variants for OA risk are summarized above.

Reference (PubMed ID)	OA joint site	Variant studied; nearest gene	OA risk	Sample size	Population	Study type
31664512	OA	rs2275913; IL17A rs763780; IL17F	A (minor) rs2275913 and C (minor) rs763780 detrimental for KOA not HOA	2,214 cases 2,474 Cx	various	Meta-analysis
31713648	KOA	rs2228611, rs2228612; DNMT1 rs2424913; DNMT3B	C (minor) rs2228611 and C (minor) rs2228612 protective: C (minor) rs2424913 detrimental	244 cases 244 Cx	Mexican mestizo	Candidate gene
32103374	KOA	rs4867568; LSPIP rs143383; GDF5; rs1558902; FTO	T (minor) rs4867568 protective: C (minor) rs143383 protective: rs1558902; FTO - no association	532 cases 927 Cx	Chinese (Han)	Candidate gene
32144005	KOA	rs2234693; ESR1	T (major) detrimental	1,033 cases 920 Cx	Chinese (Han)	Candidate gene
32250658	KOA	rs3740199, rs1871054; ADAM12 rs2073508; TGFB1	No association	132 cases 164 Cx	Mexican	Candidate gene
32364812	KOA	rs217727, rs3741219; H19 rs7158663; MEG3	A (minor) rs217727 & A (minor) rs7158663 detrimental	230 cases 230 Cx	Chinese (Han)	Candidate gene
32452514	HOA	rs10896015; LTBP3	A (minor) protective	884 cases 1896 Cx	Chinese (Han)	Candidate gene
32460535	KOA	rs10817595; AKNA	A (minor) detrimental	824 cases 1,676 Cx	Chinese (Han)	Candidate gene
32537881	HOA	rs763780; IL17F	C (minor) detrimental	796 cases 1854 Cx	Chinese (Han)	Candidate gene
32557253	KOA	rs4884, CKM	G (minor) protective	87 cases 107 Cx	Mexican	Candidate gene
32596320	OA	rs1799750; MMP1/WTAPP1	No general OA role, but association with temporomandibular joint	1,245 cases 1,230 Cx	various	Meta-analysis
32681364	KOA	rs10116772, rs7045410, rs7032713; GLIS3	Minor alleles (A, G, G) protective	810 Cases 900 Cx	Chinese	Candidate gene
32793194	KOA	rs7097780; IL15RA	G (minor) symptomatic KOA	Symptomatic 403 vs asymptomatic 148	UK	Candidate gene
32928309	KOA	rs143383; GDF5	C (minor) protective - Caucasian only	7997 KOA 12684 Cx	Caucasian/Asian	Meta-analysis
33036557	KOA	rs12700386; IL6	C (minor) detrimental. C allele increases peripheral blood IL-6 expression	352 cases 411 Cx	Chinese (Han)	Candidate gene
33055079	Hand OA	rs10916199; SNAP47	A (major) detrimental – predicted causal gene WNT9A	8,691 discovery participants. 1,203 replication	Dutch discovery US replication	GWAS
33112260	OA	rs4946936; FOXO3 rs11769597; IGFBP3	rs4946936 minor allele (T) - protective male HOA: rs11769597 - C - detrimental female KOA	337 cases 456 Cx	Dutch	Candidate gene
33181041	KOA	rs2862851; TGFA	T (minor) detrimental	392 cases 808 Cx	Chinese (Han)	Candidate gene
33590413	KOA	rs2910164 and rs57095329; MIR146A	No association	310 cases 379 Cx	Mexican (mestizo)	Candidate gene
33861510	hand OA and KOA	rs143383; GDF5 D-repeat; ASPN	C (minor) rs143383 protective; ASPN D14 allele protective	100 cases 100 Cx	Kurdish	Candidate gene
33981765	OA	rs28929474; SERPINA1 Z allele	Z allele protective	∼16,000 heterozygous vs ∼300,000 Cx		Phe-WAS
34137553	OA	rs12901499; SMAD3	mild association	2,403 cases 3,209 Cx	various	Meta-analysis
34145804	KOA	rs3819089; MMP13 rs3740199 and rs1871054; ADAM12 rs4747096; ADAMTS14	No association	150 K-L ≥ III Cases; 150 K-L ≤ II Cx	Turkish	Candidate gene
33640581	OA	Various; Cu; Zn	High Cu/Zn detrimental	N/A		Mendelian randomisation

∗HOA = hip OA, KOA= Knee OA, Cx = Control, K-L= Kellgren–Lawrence Grade. GWAS = genome-wide association study, Phe-WAS= Phenome-wide association study.

In a Phenome-wide association study using data from the UK Biobank heterozygosity the Z allele (rs28929474) of *SERPINA1*, homozygosity for which causes the rare condition α_1_-antitrypsin (AAT) deficiency, was associated with several musculoskeletal phenotypes, including a reduced risk of OA, but an increased risk of osteoporosis and lower bone mineral density (BMD)^8^. AAT can inhibit RANKL-induced osteoclast formation and bone resorption. Since heterozygosity for the Z allele reduces AAT levels, this could possibly explain both the lower BMD and associated OA risk. Recently, neutrophil elastase, for which AAT is an inhibitor, has been shown to fully activate MMP13, the major collagenase that promotes the irreversible destruction of cartilage collagen in OA^9^. Active MMP13 can proceed to inactivate AAT, thus AAT may play a complex role in multiple musculoskeletal tissues in OA.

GLIS3 is a transcriptional regulator which modulates hedgehog signalling, a pathway involved in limb development and OA severity^10^, but the factor itself is better associated with neonatal diabetes and congenital hypothyroidism, though single nucleotide polymorphisms (SNPs) around *GLIS3* are associated with OA susceptibly^11^. This year Zhang *et al.* confirmed a similar OA association (rs10116772, rs7045410, rs7032713) in a Chinese population^12^. By correlating genotype with cartilage gene expression from 200 KOA patients the minor alleles of the same SNPs were associated with significantly lower *GLIS3* expression, but higher expression of a non-coding *GLIS3* nested transcript, *GLIS3-AS1*.

The IL-17 cytokine family comprises of six protein members (A-F). Studies are contradictory as to whether genetic polymorphisms in *IL17A* and *IL17F* are associated with OA. To resolve this Lee and Song conducted a meta-analysis of eight studies and concluded that rs2275913 in *IL17A* and rs763780 in *IL17F* are associated with general OA, but the latter not with hip OA (HOA)^13^. However in a more recent study, rs763780 was associated with HOA in a Han Chinese population^14^, indicating that perhaps ethnicity is partially responsible for the contradictory associations of *IL17* genes with OA.

The main comprehensive GWAS within this year concentrated on hand OA and identified a novel risk locus centred upon the SNP rs10916199^15^. This study is particularly compelling as the authors stratified their cohort into three hand OA endophenotypes, based upon hierarchical clustering of radiograph-defined Kellgren and Lawrence grade across all 32 joints of both hands. This generated three hand OA phenotypes for analysis, essentially hand, finger and thumb. They then conducted their GWAS on each of the three hand OA phenotypes in the same cohort (∼8,700 individuals) with follow up in a replication cohort (∼1,200 individuals). This identified four OA associated loci, though only three were also significantly associated in the replication cohort. Two of these were novel (rs10916199 and rs2070852) and both associated with the thumb endophenotype. Of these rs10916199 was the most significant locus and is located on chromosome 1 near the *ZNF678*, *WNT3A* and *WNT9A* genes. To prioritise the causal gene the authors used human OA cartilage expression and methylation quantitative trait loci (eQTL and meQTL) datasets, and intersecting GWAS signals with accessible chromatin regions (ATAC-seq peaks) of human fetal cartilage and 3D chromatin conformation data from human mesenchymal stem cells (MSCs). These data, together with differential expression analysis between OA lesioned and preserved cartilage, identified *WNT9A* as the likely gene linked to rs10916199. Taken altogether, this paper demonstrates the power of assessment of stratified phenotypes in OA and that using such an approach could improve GWAS statistical power and provide novel insights into OA biology.

Although testing OA risk variants using candidate gene approaches, particularly with regards different ethnicities, can be useful these studies frequently use relatively few patients and controls. Therefore, the inclusion of diverse, non-European lineage, populations in GWAS OA studies is likely to be a productive way to identify novel OA risk variants and identify common pathogenic mechanisms across the globe^16^.

For a comprehensive summary of the current knowledge from GWAS of 124 OA-associated SNPs, encompassing 95 independent loci, we point the reader to a review article published during this year^17^.

### Functional genetic analysis

This year several studies performed detailed functional studies of OA risk variants (Table II). As mentioned, an article published this year, but discussed in last year's review, elegantly described the functional characterization of the polymorphism rs6060369 which resides in an intron of *UQCC1* yet is an enhancer variant controlling *GDF5* expression, required for synovial joint formation and maintenance. The articles hypothesis is that during human evolution variants arose as we adapted to the biomechanical demands of bipedalism. However, although these variants had a positive role in limb formation, because of antagonistic pleiotropy, some, such as those in GDF5, are also deleterious to the long-term health of the joint, resulting in OA^7^.

**Table II:** Studies from April 12,020 to April 30 2021 performing functional analysis of genetic variants for OA risk are summarized above

OA Association SNP	Nearest ORF	Variant location	Identified Causal/regulated gene	Reference (PubMed ID)
rs11583641	*COLGALT2*	3′UTR	*COLGALT2*	33760386
rs10916199	*ZNF678*	Intronic	*WNT9A*	33055079
rs11780978	*PLEC*	Intronic	*PLEC*	32580029
rs75621460	*TGFB1*	Intergenic	*TGFB1*	33760378
rs6516886	*RWDD2B*	Intergenic	*RWDD2B*	32755071
rs77245812	*MATN3*	ORF T303M (mouse T298M)	*Matn3*	33227438
rs6060369	*UQCC1*	Intronic	*GDF5/Gdf5*	32220312

Mutation T303M of matrillin-3 was originally linked to hand OA in an Icelandic cohort^18^, though in other ethnicities these findings are only partially confirmed, though additional linkage to spinal disc generation is observed^19^. Seifer *et al.* generated a mouse model carrying the equivalent mutation (mouse T298M) in matrillin-3 and extensively characterized the animals. Developmentally the mice were essentially normal and though the human mutation is associated with hand OA no similar consequence was observed in the forepaw, perhaps due to species specific differential usage and mechanical forces. The cartilage of the mutant mice contained collagen fibrils with increased diameter compared to wild type (WT), increasing the compressive stiffness of the tissue. Despite this altered cartilage property, the mutant mice showed no increase in spontaneous OA severity with age but did for surgically-induced (meniscectomy) post-traumatic OA, suggesting the matrillin-3 T298M knock-in mouse could represent a model for investigating the pathogenic mechanisms involved in OA development^20^.

A T > A SNP at rs6516886, located in a region containing multiple genes, is associated with both KOA and HOA^21^. Parker *et al.* demonstrated that the OA-associated T allele of rs6516886 correlated with increased DNA methylation of neighbouring CpGs^22^. Differential allelic expression was identified for 3 genes in proximity to the SNP, but most notably for the gene *RWDD2B*, an RWD domain–containing protein coding gene of unknown function, across multiple joint tissues, with additional evidence of correlation with local CpG methylation. Finally, the authors were able to establish that experimental reduction of this CpG methylation by overexpression of a deactivated-(d)Cas9-Ten-eleven translocation methylcytosine dioxygenase 1-(TET1) fusion protein resulted in increased expression of *RWDD2B*.

Reported as an OA risk signal, the G>A polymorphism rs75621460 is positioned between *CCDC97* and *TGFB1* on chromosome 19^23^. *TGFB1* of course encodes a ligand of the TGF-beta superfamily, with the pathway well described to regulate cartilage development and tissue homeostasis^24^. Rice *et al.* demonstrated differential enhancer activity and protein binding per allele^25^. Clustered regularly interspaced short palindromic repeats (CRISPR)-Cas9-mediated deletion of the locus implied regulation of only *TGFB1*, and correlation between DNA methylation of CpGs adjacent to the rs75621460 and *TGFB1* expression were found, especially in cartilage tissue. Targeting methylation to the locus by a DNA methyl transferase 3a (DNMT3a)-dCas9 fusion reduced enhancer activity and *TGFB1* expression, but in an allele-independent manner, implying downstream regulation potentially in response to allele-specific protein complex binding.

Similarly, Kehayova *et al.* examined HOA risk SNP rs11583641, located in the 3′UTR of *COLGALT2*, which encodes a β-galactosyltransferase that transfers β-galactose to hydroxylysine residues of collagen, identifying correlation between allelic expression and methylation of an upstream enhancer^23^^,^^26^. Experimental loss- and gain-of methylation again directed by dCas9 fusion transcriptional regulators modulated *COLGALT2* expression, however there was variable allele-related expression and it remains unclear what impact the variation in the 3′UTR sequence might have directly on post-transcriptional regulation.

With the intention of improving functional gene discovery in OA, Butterfield *et al.* developed a three-mode imaging pipeline to comprehensively phenotype the mouse knee joint and its disease-related changes^27^. Iodine-contrast-enhancer (ICE) μCT determines several cartilage and bone volume, thickness and density characteristics, joint surface replication (JSR) quantifies cartilage surface damage, and subchondral bone X-ray microradiography (scXRM) determines mineral density. 100 WT mice were used to generate a baseline reference dataset for a phenotypically normal joint, which was then validated and contrasted against joints following OA induction by destablisation of the medial meniscus (DMM) surgery.

To establish the utility of the pipeline and identify genes associated with OA, the authors characterised 50 randomly selected mutant mouse lines generated by the international mouse phenotyping consortium. Surprisingly, 50% of the lines exhibited at least one phenotypic abnormality identified by the multi-modal imaging pipeline, suggesting many genes can impact the development and homeostasis of the joint. A thorough data integration strategy was taken to prioritise these identified genes, including data from knockout phenotypes, joint tissue expression, association with human disease and previous literature, which in this case identified *Pitx1*, *Bhlhe40*, *Sh3bp4* and *Unk*. Having demonstrated the power of their joint phenotyping strategy three additional applications of the imaging pipeline were presented. Firstly, eight knockout mouse lines for genes differentially expressed in human OA were interrogated, of which joint phenotypes in 6 (*Unk*, *Josd1*, *Gsdme*, *Arhgap30*, *Ccdc6*, *Col4a2*) were found. Secondly, a comparison between 4- and 12-month-old mice identified age-related OA joint changes. Finally, a mutant *Dio2* mouse, generated to reflect an OA-associated human polymorphism (rs225014), was examined and joint differences identified compared with the WT allele. The authors propose uptake of this novel joint phenotyping strategy by the OA research community, with its advantages over the traditional Osteoarthritis Research Society International (OARSI) method of damage scoring including increased sensitivity, and reduced cost and time, albeit requiring a large investment for the imaging facilities. Large scale screening of mutant lines for joint phenotypes (i.e., human OA GWAS hits) could provide data with reduced bias than researcher selected gene studies and provide valuable negative data for use in computational expansion of existing knowledge of joint damage associated genes^28^.

## Transcriptomics and proteomics

Much of the existing OA genomics data is from cartilage tissues. This year saw several genomics studies examining other tissues of the joint and investigating the relationships between joint tissues. Tuerlings *et al.* examined subchondral bone using RNA sequencing (RNA-seq) to highlight the differentially expressed genes between paired preserved and lesional regions in 18 KOA and 6 HOA patients^29^. Comparison to articular cartilage revealed shared altered genes between joint sites, including the OA susceptibility genes *IL11* and *CHADL*, which are candidates for future studies in *in vivo* OA models. However, genetic risk genes themselves may not represent the most drug tractable targets within an implicated signalling pathway. A future challenge from data such as this is to use this knowledge of the processes altered to gain a more mechanistic insight into what is driving them^30^.

In a multi-tissue omics study, Steinberg *et al.* performed both transcriptomics and proteomics of cartilage and synovium tissues as well as exome genotyping from peripheral blood in 115 OA patients^31^. The authors identified *cis* eQTLs for 1891 genes and 38 *cis* protein-eQTLS. The authors demonstrate the utility of this molecular map in determining the effector genes for non-coding GWAS lead variants. Statistical co-localisation of five OA GWAS signals were found with the molecular QTLs (*ALDH1A2*, *NPC1*, *SMAD3*, *FAM53A* and *SLC44A2*), two of which are not the nearest genes to the lead variant. Comparison of the high-grade and low-grade OA cartilage using transcriptomics and proteomics revealed 2,557 and 2,233 differentially abundant genes and proteins, respectively, of which 409 were dysregulated at both regulatory levels. The authors computationally screened for drug repurposing opportunities by testing for enrichment of the 148 upregulated proteins in damaged cartilage in a library of gene expression changes from *in vitro* drug treatments. The top enriched drugs such as IB-MECA (CF 101, Piclidenoson), VEGF-receptor-2-kinase-inhibitor-IV and nornicotine could potentially reduce the expression of the upregulated OA damaged cartilage signature. If these upregulated genes are detrimental in OA these drugs would be predicted to improve the cartilage phenotype and could potentially be repurposed to treat OA.

An important consideration for any future drug treatment in OA is the heterogeneity of the disease. Building on previous attempts to stratify OA patients with molecular data^32^ Steinberg and colleagues also assessed gene expression heterogeneity across a cohort of 113 KOA patient cartilage and synovium samples^33^. Patient subgroups were identified in both low-grade (intact) cartilage and synovium with the predominant discriminator being inflammation, although the patient subgroups in each tissue were independent of each other. A seven gene classifier was identified that could distinguish the subgroups and the corresponding main axis of molecular heterogeneity. Importantly, the classifier was verified in an independent validation cohort. An important future direction is translation of these tissue-based classifiers to more clinically assayable synovial fluid or serum-based biomarkers to enable longitudinal studies or examine differential response to putative therapeutics between the identified molecular subgroups.

Synovial fluid acts as a medium for joint tissue crosstalk with proteins such as growth factors, chemokines and cytokines secreted from the cartilage and synovium that may contribute to joint damage. The relative role of the synovium in driving OA remains unclear. Chou *et al.* applied single-cell sequencing to examine the crosstalk between matched synovial and damaged and intact cartilage tissue^34^. Using unsupervised clustering they identified 12 clusters of cells types in the synovium and 7 chondrocyte cell states. Detectable expression (>1% cells) of the commonly studied cytokines in OA such as *TNF*, *IL6* and *IL1B* was restricted to synoviocytes, supporting previous cartilage RNA-seq based evidence that chondrocytes do not express these mediators^32^. In contrast, growth factors were expressed by both cell types and were more abundant in the synovial fluid than the cytokines. Future comparison of OA samples to non-OA controls to establish the healthy range and expression of mediators in the synovial fluid and tissues would assist in prioritising those proteins likely to be pathogenic. This work offers the potential for an assay or target against a particular cell population for monitoring progression or outcome of treatment. For instance, a subset of chondrocytes, enriched in damaged cartilage, were responsible for much of the cartilage protease expression.

The identification of the upstream regulators of the differentially expressed genes in each cell type in the Chou *et al.* study provides an insight into the communication between the synovial expressed cytokines and the chondrocytes^34^. Computational prediction of upstream drivers of the observed differential expression is challenging. There is risk of bias in their prediction due to the popularity of the regulators in the literature^35^ and these prediction approaches, as in the *in silico* drug screen described^31^, use data derived primarily from non-joint cell types so only conserved regulatory mechanisms and drug responses are likely to be identified. A study of the transcriptional responses to the cytokines and growth factors in joint tissues would provide useful information for computational inference of upstream regulators in the joint. For example, Mimpen *et al.* examined the transcriptional response of synovial fibroblasts and chondrocytes to members of the IL17 family, previously shown to be detectable in the synovial fluid of a subset of OA patients and, as described, genetically linked to the disease^36^. IL17A induced differential expression of genes, such as *IL6*, *NFKBIZ*, *SOD2* and *ZC3H12A* which are both differentially expressed in human OA and alter joint phenotype in OA animal models^37^, ^38^, ^39^, ^40^. Data such as these could potentially allow more accurate inference of the main synovial fluid-based drivers of differential expression in affected tissues.

Transcriptomics of articular cartilage from mouse joints is technically challenging due to both contamination of dissected tissue with other cell types and the potential requirement for a long enzymatic digestion due to the extracellular matrix (ECM) rich nature of the cartilage. Sunkara *et al.* present a joint tissue cutting strategy where the distal section of the femur was segmented into three parts with known tissues and cell populations^41^. Importantly, treatment of the cells with the transcriptional inhibitor actinomycin-D during the ECM digestion revealed alterations in chondrocyte marker genes such as *Col2a1*, *Sox9*, and *Comp*, suggesting digestion itself can alter the measured chondrogenic phenotype of the studied cells. Single cell RNA-seq of the articular cartilage containing segment allowed the identification of a pure chondrocyte transcriptome from a small number of cells which when combined with bulk RNA-seq of the segments generated a high confidence chondrocyte gene signature. This chondrocyte signature could be used in computational deconvolution of both existing and future impure dissected articular cartilage bulk-RNA-seq into mixtures of cell types, to study chondrocyte-specific transcription responses^42^.

## Epigenetics

### DNA methylation

This year there were few new studies of global DNA methylation in OA, with an exception being in Kashin–Beck disease^43^. As discussed, several studies focused on OA genetic association loci correlating with different patterns of DNA methylation, meQTLs. Smeriglio *et al.*^44^ built upon the group's previous implied role of 5-hydroxymethylcytosine (5hmC) in OA, where levels increased at OA-associated genes in cartilage^45^, by showing an accumulation of 5hmC during DMM-induced OA in mice which corresponded with gene expression changes. 5-methylcytosine (5mC) oxidation to 5hmC is the first step in DNA demethylation, which can be mediated by the methylcytosine dioxygenase TET1. TET1 knockout (KO) mice following DMM surgery had reduced cartilage damage and osteophytes. Importantly, the therapeutic potential of targeting TET1 was investigated with frequent injections of TET inhibitor 2-HG post-DMM surgery in WT mice, the outcome being to phenocopy the effect of genetic ablation of TET1. The authors state that TET1-specific inhibitory peptides have recently been developed, opening the possibility for improved preclinical studies to establish the potential of this approach to protect against OA progression. However, TET1 acts as a tumor suppressor gene and TET1 inhibition is linked to cancer suggesting systemic delivery would be undesirable^46^.

### Non-coding RNA

**Fig. 2:**
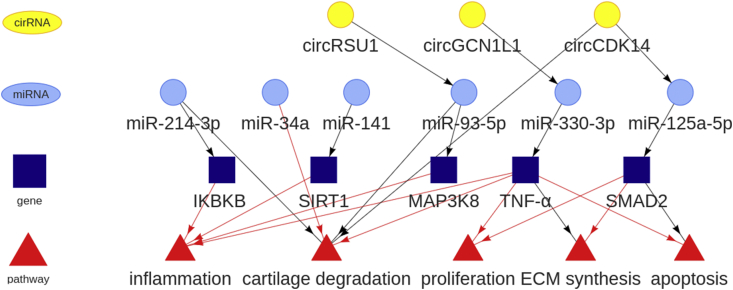
**Targets and pathways of the selected non-coding RNAs studied in OA this year.** The reported downstream signalling and interactions were extracted from the non-coding RNA papers reviewed within the main text^52^^,^^55^^,^^60^, ^61^, ^62^^,^^74^. Pathways are illustrated as red triangles, circRNA are yellow circles, microRNAs are light blue circles and gene/protein expression changes are denoted in dark blue squares. Black and red arrows indicate negative and positive regulation, respectively. Plotted using VisNetwork (v 2.0.9).

This year saw a huge number of studies evaluating non-coding RNA, particularly microRNAs in OA (Fig. 1, Fig. 2). There was also increasing interest in the concept of competing endogenous RNAs (ceRNAs) which includes circular RNAs (circRNAs) and long non-coding RNAs (lncRNAs), which act as microRNA sponges, though the physiological relevance of ceRNAs is questionable^47^. CircRNAs are highly stable transcripts produced as a back splicing event often from open reading frame transcript exons, while lncRNAs are defined as transcripts exceeding 200 nucleotides in length that are not translated into protein. For the first time, SnoRNAs altered in OA were profiled and several were studied through loss and gain of function experiments^48^. This year also saw the first study of tRNA-derived fragments (tRFs) in chondrocytes which have been ascribed microRNA-like functions leading to target mRNA repression^49^.

Many of the non-coding RNA studies were performed in *in vitro* chondrocyte cell lines under inflammatory (lipopolysaccharide (LPS) or IL1 treatment) conditions and generally present a linear cascade (axis) of signaling downstream of the studied RNA to inflammation or ECM degradation via a single target (Fig. 2). Robust high-throughput identification, validation and understanding of the interplay of the likely hundreds of targets of each non-coding RNAs remains challenging and is further exacerbated by complexities such as isomiRs, variants of microRNAs which can alter their target repertoire^50^.

### MicroRNA

The difficulties of accessing joint tissues has attracted research into the possibility of delivering therapeutics into cartilage. Liang *et al.* sought a solution to this ‘grand challenge’ with the use of exosomes to deliver microRNAs into chondrocytes^51^. Utilising a previously identified chondrocyte-affinity peptide (CAP) fused to a lysosome membrane protein the authors were able to derive exosomes able to selectively deliver miR-140 into human chondrocytes, but not MSCs, and cause suppression of miR-140 targets *ADAMTS5* and *MMP13*. Articular injection of fluorescently labelled miR-140-containing exosomes into rats indicated that the CAP-exosomes could penetrate cartilage *in vivo* and offer protection against cartilage damage in the DMM model of OA.

Ji *et al.* in a highly comprehensive study described the functional assessment of miR-141/200c cluster in OA^52^. These two microRNAs are co-expressed from the *MIR200CHG* gene on chromosome 12 (*Gm15884* on chr6 in mouse). In a screen of differentially expressed microRNAs in end-stage OA cartilage, both microRNAs were upregulated correlating with hypomethylation of *MIR200CHG* gene promoter. Previously the same group, using gain- and loss-of-function studies in mouse models, showed that miR-141 was a key regulator of intervertebral disc degeneration, in part via regulating the deacetylase SIRT1 levels^53^. In the current work the group described a similar function of miR-141 in growth plate and articular cartilage chondrocytes. Adult miR-141/200c conditional knock-out (cKO) mice, either aged or subjected to OA-inducing DMM surgery, had reduced cartilage damage, indicating that the microRNAs are catabolic regulators of the process driving OA. The authors then performed detailed further work to deliver miR141/200c mimics and inhibitors to cartilage. Firstly, the cell-SELEX (systematic evolution of ligands by exponential enrichment) method^54^ was used to select a DNA aptamer, termed tgg2, which selectively bound to chondrocytes, potentially via Fibroblast Growth Factor Receptor 1 (FGFR1). This was conjugated with a dendritic polymer to create a stable nanoparticle capable of penetrating human OA cartilage explants *ex vivo*. Finally, delivery of miR-141/200c mimics or inhibitors using the nanoparticles via repeated intra-articular injection exacerbated or reduced DMM-induced OA damage, respectively. The proposed mechanism of how this occurs is via miR-141/200c regulation of IL-6 mediated inflammatory signalling, through the direct deacetylation of IL-6 by the miR-141 target SIRT1 (Fig. 2).

Endisha *et al.* investigated the role of miR-34a-5p in OA having previously identified its upregulation in late-stage OA synovial fluid^55^. After confirming the increase in miR-34a-5p in both human and experimental KOA, treatment of both OA chondrocytes and synoviocytes with miR-34a-5p mimic and inhibitor indicated the reciprocal regulation of OA anabolic and catabolic marker genes such as *COL2A1* and *MMP13* (Fig. 2). Importantly, these functional experiments were replicated *in vivo* where three fortnightly injections of a miR-34a-5p inhibitor reduced cartilage damage following DMM surgery in mice, while injection of miR-34a-5p mimic promoted spontaneous OA development. The authors also demonstrated that injection of miR-34a-5p inhibitors could suppress OA in a high fat diet-accelerated DMM model. MiR-34a-5p genetic ablation confirmed protection against DMM-induced cartilage damage while RNA-seq of the cartilage from the animals identified potential disease-relevant targets.

From the same group, Ali *et al.* characterised circulating microRNAs from the plasma of patients with early and late radiographic KOA, identifying a panel of microRNAs which differentiated between the two groups^56^. This adds to a small number of studies which have assessed circulating microRNAs in OA, but there currently appears little consensus in the microRNAs identified, potentially owing to differences in patient cohorts, study design and technological methodology utilized^57^.

Finally, Cao *et al.* identified reduced levels of miR-214-3p in human and mouse KOA cartilage. miR-214-3p inhibited the IL-1-mediated activation of the catabolic gene expression program in chondrocytes, potentially by directly targeting the NF-κB pathway kinase *IKBKB* to suppress NF-kB signalling. Intra-articular delivery of miR-214-3p mimic or inhibitor was able to respectively suppress or promote cartilage degradation in the DMM-induced OA mouse model, although only small numbers of mice were used and no evidence of alterations to miR-214-3p or *IKBKB* levels were presented (Fig. 2).

### Long non-coding RNAs

To rationalize the lncRNAs most important in OA, van Hoolwerff *et al.* established lncRNA-mRNA co-expression networks using paired (lesion vs intact cartilage) RNA-seq data from almost 100 OA patients^58^. This identified 5,053 robustly expressed lncRNAs, of which 191 were significantly differentially expressed. These lncRNAs, which are predominantly intergenic or antisense transcripts, may regulate differentially expressed mRNAs in *cis* in cartilage.

Using RNA-seq of synovial fibroblasts Nanus *et al.* sought to profile the lncRNA changes in obesity-associated OA^59^. Nineteen lncRNAs were differentially expressed in fibroblasts of obese compared to normal-weight HOA patients, including the lncRNA *MALAT1*. Synovial fibroblasts from the obese group exhibited an inflammatory phenotype, which may, the authors conclude, be partially mediated by *MALAT1*.

### CircRNAs

Yang *et al.*, having demonstrated the increase in oxidative stress with age in OA, identified expression changes in 550 circRNAs following 5 days of H_2_O_2_ treatment of chondrocytes to induce oxidative stress^60^. The most upregulated was *circRSU1*, and experimental manipulation of its levels altered the balance of chondrocyte gene expression towards a catabolic profile with increased metalloproteinase expression and reduced matrix gene expression. The authors demonstrated that *circRSU1* exhibited ceRNA activity against several miRNAs but most notably miR-93-5p. Remarkably, *circRSU1* intra-articular delivery induced the same severity of OA in mice as DMM surgery, while mutation of the miR-93-5p binding site in the circRNA completely abrogated this effect. Finally they established a mechanism where miR-93-5p targets *MAP3K8* thereby suppressing extracellular signal-regulated kinase (ERK) and NF-κB signalling, a scenario that would be reversed by the experimental or age-related upregulation of *circRSU1* and the concomitant reduction in available miR-93-5p.

Further notable circRNA studies in the OA field briefly include, *circGCN1L1* in the progression of OA in rats by acting as a sponge for miR-330-3p which targets TNF-α, thereby promoting synoviocyte proliferation and chondrocyte apoptosis^61^, and *CircCDK14*, which interacts with miR-125a-5p interfering with its repression of *SMAD2*, protecting against OA in rabbits by regulation of ECM and chondrocyte viability^62^ (Fig. 2).

## Future approaches and summary

Driven by the decreasing costs of single cell technology and improvements in cell isolation techniques we expect to see growth in multimodal single cell technology applied to OA research. For example, ATAC-seq and RNA-seq could be performed simultaneously to gain greater insight into the transcriptional regulation in cell states in OA. Pooled CRISPR screening approaches with single cell read-out offer the opportunity characterize the function of hundreds of potential OA effector genes in relevant cell types simultaneously^63^. Another possible future sequencing approach is spatial RNA-seq which allows transcriptomics from intact histological sections while retaining valuable spatial tissue information^64^^,^^65^. Probabilistic methods could be used to integrate existing single cell data with spatial RNA-seq data to explore the spatial relationships between previously identified cell types and states^66^. This would allow investigation of the spatial distribution of single-cell seq identified cell types and give insights into the relative accessibility of these cells to synovial fluid mediators or any planned intra-articular interventions.

OA is increasingly seen as a complex heterogenous syndrome affecting the whole joint structure, not just cartilage. There is also limited synovial inflammation but to varying degrees for each patient. The heterogenous nature of the disease may partly explain the number and impact of GWAS-identified loci, which only explain a small proportion of the phenotypic variance^23^. Current GWAS have included thousands of patients with OA^21^^,^^23^^,^^67^, but a full meta-analysis of multiple populations will likely lead to the identification of further risk variants. This year Boer *et al.*, elegantly demonstrated how analysis of well-characterized patient cohorts, where patients were segregated into distinct disease sub-types (in this case of hand OA), could increase the discovery power of a relatively modestly sized GWAS^15^. An expectation in the coming year(s) would be that further patient or disease stratification could improve GWAS-derived knowledge of disease-associated loci for OA of the other joint sites. Clinical adoption of stratification approaches using other molecular data such as global DNA methylation patterns^68^, ^69^, ^70^ and tissue RNA-seq as described this year^33^ and attempted previously^32^ are impeded by joint tissue inaccessibility. An integrated approach of molecular tissue characterization combined with assayable biomarker measurement such as from synovial fluid^71^ or circulating microRNA^56^, may help translation of these approaches and clarify if such genomically stratified patient groups are of clinical utility. Several new disease modifying therapies for OA are still being investigated in ongoing clinical trials and it will be of interest to speculate if these treatments could be improved if targeted to a more specific disease subtype^72^.

## Contributions

All authors were involved in the design of the review, and analysis and interpretation of studies included. JS and DAY assessed the manuscripts to include. All authors drafted the article, and all approved the final version to be submitted.

## Conflict of interest

All authors declare no conflicts of interest.
